# Herpes Simplex Virus Hepatitis: An Overlooked Cause of Acute Liver Failure Across a Broad Spectrum of Immunocompromise

**DOI:** 10.1111/liv.70756

**Published:** 2026-06-21

**Authors:** Mario U. Mondelli, Serena Ludovisi, Angela Di Matteo, Gülşen Özkaya Şahin, William Irving, European Society of Clinical Microbiology and Infectious Diseases Study Group for Viral Hepatitis (ESGVH)

**Affiliations:** ^1^ Department of Internal Medicine and Therapeutics University of Pavia Pavia Italy; ^2^ Division of Prevention and Vaccine Centres ASST Santi Paolo e Carlo Milan Italy; ^3^ Division of Infectious Diseases, Department of Medicine Fondazione IRCCS Policlinico San Matteo Pavia Italy; ^4^ Clinical Microbiology, Infection Prevention and Control Office for Medical Services, Region Skåne Lund Sweden; ^5^ Division of Medical Microbiology, Department of Laboratory Medicine Lund, Medical Faculty Lund University Lund Sweden; ^6^ University of Nottingham and Nottingham University Hospitals, NHS Trust Nottingham UK

**Keywords:** acute liver failure, acyclovir, hepatitis, herpes simplex virus

## Abstract

**Background and Aims:**

Herpes simplex virus (HSV) infection is common worldwide, but hepatic involvement is rare. HSV hepatitis is an uncommon yet frequently catastrophic cause of acute hepatitis and acute liver failure (ALF), accounting for < 1% of ALF cases while carrying very high mortality when diagnosis and antiviral therapy are delayed. Although classically associated with profound immunosuppression and late pregnancy, HSV hepatitis is increasingly recognized in patients with less obvious or “non‐traditional” forms of immune dysfunction.

**Methods:**

We describe three cases of HSV hepatitis presenting with distinct risk profiles and clinical manifestations and review the diagnostic and therapeutic challenges associated with this condition.

**Results:**

Clinical presentation was nonspecific in all cases, and mucocutaneous lesions were absent or delayed, contributing to diagnostic uncertainty. All patients exhibited marked aminotransferase elevation with relatively modest hyperbilirubinemia (“anicteric hepatitis”), frequently accompanied by cytopenias and coagulopathy. Delayed recognition was associated with rapid clinical deterioration, whereas earlier initiation of intravenous acyclovir was associated with biochemical and clinical improvement. Diagnosis was established using polymerase chain reaction–based detection of HSV DNA in blood and tissue samples.

**Conclusions:**

HSV hepatitis remains an under‐recognized cause of ALF across a broad spectrum of immunocompromised and seemingly immunocompetent patients. Given the narrow therapeutic window and favourable safety profile of acyclovir, empiric intravenous antiviral therapy should be considered in patients with ALF of indeterminate aetiology while diagnostic testing is pending, particularly in high‐risk clinical settings. Increased awareness of this overlooked diagnosis may improve outcomes and prevent avoidable mortality.

## Key Points

- HSV hepatitis is rare but highly lethal and should be considered in all cases of acute hepatitis or acute liver failure of unclear aetiology. Absence of skin or mucosal lesions does not exclude the diagnosis.
- Marked aminotransferase elevation with minimal bilirubin increase (“anicteric hepatitis”), especially when accompanied by fever or cytopenias, is a key diagnostic clue.
- Early antiviral therapy is the most important determinant of survival. Long‐term antiviral suppression is generally indicated following recovery, particularly in immunocompromised patients.

## Lay Summary

Herpes simplex virus (HSV), best known for causing cold sores and genital herpes, can rarely infect the liver and lead to severe liver failure, even in people without obvious immune system problems. Because symptoms are often nonspecific and typical HSV skin lesions may be absent, diagnosis is frequently delayed, but early treatment with the antiviral drug acyclovir can be life‐saving. We highlight here the need for clinicians to consider HSV as a possible cause of unexplained acute liver failure and to start antiviral treatment promptly while awaiting test results.

## 1 Introduction

Herpes simplex virus (HSV) infection is ubiquitous worldwide, with HSV type 1 (HSV‐1) and type 2 (HSV‐2) establishing lifelong latency in sensory neurons after primary infection. Although HSV most commonly causes mucocutaneous disease, systemic dissemination may occur, particularly in the presence of impaired or dysregulated host immunity, leading to severe organ involvement including hepatitis [1, 2, 3, 4].

HSV hepatitis is thought to result from massive viral replication within hepatocytes and sinusoidal endothelial cells, producing extensive focal or confluent hepatic necrosis. Histopathological studies demonstrate haemorrhagic necrosis with characteristic viral cytopathic effects, reflecting predominantly direct cytolytic injury rather than immune‐mediated hepatotoxicity [5, 6]. Experimental models indicate that early control of HSV dissemination depends critically on intact type I interferon signalling, natural killer cell activity, and HSV‐specific CD8^+^ T‐cell responses; impairment of these pathways permits unchecked viral replication and high intrahepatic viral burden [7, 8, 9].

HSV hepatitis is a rare but highly aggressive cause of acute hepatitis and acute liver failure (ALF), accounting for < 1% of all ALF cases [2, 10]. Despite its rarity, HSV hepatitis carries a disproportionately high mortality, approaching 70%–90% when diagnosis and antiviral therapy are delayed [10, 11]. Data from the Acute Liver Failure Study Group demonstrate that HSV‐associated ALF is frequently unrecognized during life, emphasizing the ongoing diagnostic gap even in specialized centres [12].

Once considered an almost exclusive complication of profound immunosuppression—such as hematologic malignancies, solid organ or haematopoietic stem cell transplantation, or high‐dose corticosteroid therapy—HSV hepatitis is now increasingly reported in broader clinical settings [1, 2]. Pregnant women, particularly in the second and third trimesters, represent a distinct high‐risk group due to physiological alterations in cellular and humoral immunity [3, 13]. Importantly, up to one quarter of reported cases occur in individuals without overt immunosuppression, highlighting the limitations of traditional risk stratification [2, 14].

Clinical presentation is typically nonspecific and includes fever, abdominal pain, nausea, vomiting, and malaise. Classical mucocutaneous herpetic lesions are absent in more than half of cases, further contributing to diagnostic delay [12, 15, 16]. A characteristic laboratory profile—marked elevation of aminotransferases with minimal hyperbilirubinemia (“anicteric hepatitis”)—may provide an early clue but is not pathognomonic [15, 17].

Although molecular diagnostics and guideline recommendations have improved awareness, HSV hepatitis continues to be missed in real‐world practice. This reflects not only the rarity of the condition but also persistent cognitive and clinical barriers: the absence or late appearance of mucocutaneous lesions, the misleading reassurance of minimal hyperbilirubinemia, and the underestimation of immune vulnerability in patients without overt or ongoing immunosuppression. Remote transplantation, biologic immunomodulatory therapies, and other “non‐traditional” states of immune dysfunction may not be readily perceived as risk factors for disseminated HSV infection. Here, we describe three cases of HSV hepatitis spanning a broad spectrum of immune compromise, each illustrating distinct diagnostic pitfalls and therapeutic inflection points. Through these cases, we aim to highlight how delayed recognition—often driven by misattribution to drug‐induced or ischemic hepatitis—can be rapidly catastrophic, whereas timely empiric antiviral therapy can be lifesaving. By reframing HSV hepatitis as a diagnostic consideration in acute hepatitis and acute liver failure regardless of apparent immune status, this series underscores the need for earlier virological testing and a lower threshold for empiric intravenous acyclovir in high‐risk clinical contexts.

## 2 Patients and Methods

We retrospectively identified all adult patients diagnosed with HSV hepatitis at our institution between 2005 and 2015. Diagnosis was suspected based on rapidly rising liver enzymes (AST and ALT > 1000 U), signs of acute liver failure (coagulopathy and/or encephalopathy), fever, leukopaenia, normal or mildly elevated bilirubinaemia, in an immunocompromised patient.

Obvious causes like Hepatitis A, Hepatitis B, Hepatitis E, drugs, or alcohol were excluded. Diagnosis was established by detection of HSV DNA in serum samples using a quantitative polymerase chain reaction (PCR)–based assay performed according to standardized laboratory protocols. Clinical, laboratory, virological, and outcome data were extracted from medical records. The study was conducted in accordance with the Declaration of Helsinki. This retrospective case series was based on irreversibly anonymized historical clinical data. Given the impossibility of contacting the patients, a waiver of informed consent was granted by the Policlinico San Matteo Ethics Committee.

## 3 Results

### 3.1 Case Series

#### 3.1.1 Case 1

A 54‐year‐old woman with a history of acute myeloid leukaemia treated with allogeneic bone marrow transplantation 7 years earlier was admitted to the Infectious Diseases ward with fever, dysuria, and acute urinary retention. She was not receiving immunosuppressive therapy at the time of admission and had been leading an active, independent life.

Initial laboratory evaluation revealed severe hepatocellular injury (Table 1) consistent with acute hepatitis. Common causes of acute liver failure, including ischemic, toxic, and major hepatotropic viral etiologies, were promptly investigated and excluded.

**TABLE 1:** Clinical characteristics, laboratory findings, management, and outcomes of patients with herpes simplex virus hepatitis.

Characteristic	Case 1	Case 2	Case 3
Age, years/Sex	54/Female	18/Female	52/Male
Underlying condition	Remote allogeneic bone marrow transplantation (7 years prior)	Seronegative rheumatoid arthritis	Relapsed acute promyelocytic leukaemia
Immunosuppressive context at presentation	None	Adalimumab and methotrexate	Active hematologic malignancy; recent intrathecal chemotherapy and radiotherapy
HSV type	HSV‐1	HSV‐2	HSV‐1
Mucocutaneous involvement	Absent	Genital and oral lesions; palms and soles (delayed)	Severe mucositis and ocular involvement
Peak AST, U/L	13 495	2089	12 097
Peak ALT, U/L	8345	1211	N.A.
Peak total bilirubin	Mild elevation	Normal	N.A.
Cytopenias/coagulopathy	Leukopaenia, thrombocytopaenia, coagulopathy	Leukopaenia, thrombocytopaenia, coagulopathy	Coagulopathy
Time from admission to IV acyclovir (days)	2	9	10
Peak HSV DNAemia (copies/mL)	48 × 10^6^	Detected (qualitative PCR only)	191 × 10^6^
Antiviral therapy	IV acyclovir; foscarnet added	IV acyclovir	IV acyclovir; foscarnet added
Clinical outcome	Recovery; long‐term antiviral suppression	Full recovery; suppressive therapy	Death from multiorgan failure

*Note:* Summary of demographic characteristics, risk factors, laboratory findings, antiviral treatment, and outcomes in three patients with herpes simplex virus (HSV) hepatitis. All patients presented with marked hepatocellular injury and minimal hyperbilirubinemia. Diagnosis was established by polymerase chain reaction–based detection of HSV DNA in blood and/or tissue samples. In Case 2, oral acyclovir was initiated earlier at a subtherapeutic dose and is not considered effective antiviral therapy.

Abbreviations: ALT, alanine aminotransferase; AST, aspartate aminotransferase; N.A., not available.

At admission (day 0), empiric antibiotic therapy with levofloxacin was initiated, and urinary catheterization was performed. No cutaneous or mucosal lesions were observed. During history taking, the patient's husband reported recent sexual intercourse while he was experiencing active herpes labialis. Forty‐eight hours after admission, plasma testing revealed a markedly elevated HSV viral load (Table 1). Intravenous acyclovir (10 mg/kg q8h) was started on day 2. Due to a slow decline in viral load, foscarnet was added at the dose of 90 mg/kg q12 h on day 5.

Despite antiviral therapy, the patient's clinical condition and laboratory findings (Table 1) rapidly deteriorated, progressing to fulminant hepatitis. She developed leukopaenia, thrombotic thrombocytopaenic purpura, pleural effusion, pulmonary oedema, and acute heart failure requiring intensive care support. Over the subsequent 14 days, her condition gradually improved, with complete recovery of liver function.

HSV was isolated from plasma, gastric biopsy specimens, and oral and vaginal mucosal swabs, and identified as HSV‐1. Low‐level HSV DNAemia persisted for up to 46 months after hospital discharge, and long‐term acyclovir prophylaxis was maintained. The patient had documented HSV seropositivity prior to bone marrow transplantation; however, donor serologic status was unknown.

#### 3.1.2 Case 2

An 18‐year‐old woman with seronegative rheumatoid arthritis treated with adalimumab and methotrexate for 2 years presented to the emergency department with abdominal and pelvic pain. She was admitted to the Rheumatology ward with a working diagnosis of pelvic inflammatory disease and was started on intravenous ceftriaxone (1 g twice daily) and oral doxycycline (100 mg twice daily).

On the following day, vesicular lesions suspicious for genital herpes were noted, and oral acyclovir was initiated at a subtherapeutic dose. During further medical history assessment, the patient disclosed recent initiation of sexual activity. Over the next 6 days, her clinical condition worsened, with increasing abdominal pain, anorexia, weakness, nausea, and vomiting. Laboratory testing revealed leukopenia (nadir 3100 WBC/mL with relative lymphocytosis) and thrombocytopenia.

On day 8, the patient became weak and drowsy. Laboratory evaluation demonstrated severe hepatic dysfunction (Table 1), with prothrombin time reduced to 53%, while bilirubin and alkaline phosphatase levels remained within normal ranges. Drug‐induced hepatitis was suspected, and all medications—including acyclovir—were discontinued.

The following day (day 9), the patient's condition further deteriorated, with elevation of pancreatic enzymes. Physical examination showed no overt neurological impairment but revealed hepatosplenomegaly. Although genital lesions had partially resolved, new vesicular lesions appeared on the oral mucosa, palms, and soles. After Infectious Diseases consultation, swabs from cutaneous and mucosal lesions were obtained for virological analysis, and peripheral blood was tested for HSV‐1 and HSV‐2 DNA. Intravenous acyclovir (10 mg/kg q8h) was promptly initiated based on clinical suspicion of HSV hepatitis.

A rapid clinical and biochemical improvement ensued, with marked reductions in AST, ALT, and pancreatic enzyme levels, normalization of white blood cell count, and progressive correction of thrombocytopenia and coagulopathy. Within 48 h, HSV‐2 was isolated from skin lesions, and HSV DNA was detected in plasma, confirming the diagnosis. The patient showed substantial improvement by day 14 and was discharged on day 25. Oral acyclovir was continued at 400 mg five times daily until day 60, followed by long‐term suppressive therapy at 400 mg three times daily.

This episode was consistent with a primary HSV‐2 infection, as HSV‐2–specific IgG antibodies were absent 6 months prior to symptom onset, with documented seroconversion after recovery.

#### 3.1.3 Case 3

A 52‐year‐old man was admitted to the Haematology ward for central nervous system relapse of acute promyelocytic leukaemia, previously treated with allogeneic bone marrow transplantation 24 months earlier. At day 0, he received intrathecal methotrexate and panencephalic radiotherapy.

By day 10, the patient developed severe mucositis. HSV‐1 was isolated from pharyngeal and ocular swabs, and a small corneal ulcer was identified. Intravenous acyclovir (10 mg/kg every 8 h) was initiated. At that time, HSV‐1 DNAemia was 191 × 10^6^ copies/mL.

On day 13, laboratory tests revealed elevated AST (Table 1) and prothrombin time of 40%. Chest computed tomography demonstrated interstitial pneumonia with diffuse ground‐glass opacities, accompanied by oxygen desaturation. HSV DNAemia remained markedly elevated (Table 1). Foscarnet (90 mg/kg q12h) was added to antiviral therapy. Despite these measures, the patient's condition and laboratory findings rapidly worsened (Table 1). He died on day 15 due to respiratory failure and multiorgan failure.

These three cases illustrate the wide clinical spectrum of HSV hepatitis and highlight critical challenges in risk stratification, clinical recognition, diagnostic approach, and therapeutic decision‐making, which are discussed in detail below.

## 4 Discussion

The vast majority of HSV infections do not involve the liver, raising the question of why a small subset of individuals develop the catastrophic manifestation of HSV hepatitis. While viral inoculum may influence disease severity, host immune responses appear to be the dominant determinant of clinical outcome. Effective control of HSV requires coordinated innate and adaptive immunity. Indeed, deficiencies in CD4^+^ and CD8^+^ T‐cell function are associated with severe and recurrent disease, as observed in transplant recipients and other classically immunosuppressed patients [3, 18]. However, increasingly, unusually aggressive HSV infection has been linked to more subtle defects in innate immune sensing and antiviral signalling. Recognition of HSV‐derived pathogen‐associated molecular patterns by pattern recognition receptors, including Toll‐like receptors and cytosolic DNA sensors such as cGAS–STING, triggers type I interferon production and early viral containment [19, 20, 21]. Inborn errors affecting these pathways—well described in HSV encephalitis—result in impaired interferon responses and uncontrolled viral replication and may plausibly underlie cases of HSV hepatitis occurring in patients without overt immunosuppression [22, 23]. Consistent with this, HSV has evolved multiple mechanisms to subvert innate immune defences, including interference with cGAS–STING signalling and complement activation, underscoring the critical role of early antiviral immunity in preventing dissemination [24, 25, 26]. Together, these observations support the concept that susceptibility to HSV hepatitis reflects not only profound immunosuppression but also qualitative or context‐dependent failures of antiviral immune surveillance.

The traditional concept of the immunocompromised patient has evolved substantially over recent decades. Advances in oncology, transplantation, immunomodulatory therapies, and critical care have resulted in a growing population of patients with varying degrees of immune dysfunction that may not be immediately apparent. Consequently, severe viral infections such as HSV hepatitis increasingly occur in patients not classically perceived as immunocompromised. The present case series illustrates the expanding spectrum of immune dysfunction associated with HSV hepatitis and underscores the persistent diagnostic and therapeutic challenges associated with this rare but frequently lethal condition.

These cases illustrate several recurring diagnostic pitfalls: absence or delay of mucocutaneous lesions, misattribution of cytolytic hepatitis to drug toxicity, underestimation of immune dysfunction associated with biologic therapies or remote transplantation, and reliance on bilirubin elevation to signal severity.

Indeed, beyond classical states of profound immunosuppression, biologic therapies such as tumour necrosis factor‐α blockade may impair antiviral immune surveillance and facilitate disseminated HSV infection, as exemplified by Case 2. This observation is consistent with experimental and clinical evidence linking TNF‐α inhibition to impaired viral control and severe HSV disease [27]. Case 1, although a remote recipient of allogeneic bone marrow transplantation, was not receiving immunosuppressive therapy, making her immune vulnerability difficult to recognise. Case 3 represents, instead, the prototypical high‐risk host, with active hematologic malignancy and recent intensive therapy.

Norvell et al. analysed 137 adult cases of HSV hepatitis and found that while transplant recipients and patients receiving immunosuppressive therapy comprised a significant proportion, approximately 24% of cases occurred in individuals considered immunocompetent at presentation [2]. Pregnancy—particularly during the second and third trimesters—represents a distinct high‐risk condition, associated with altered cellular immunity, reduced T‐cell responses, and immunoglobulin dilution [13, 16]. A diagnosis of HSV hepatitis should be considered in any pregnant patient who has been provisionally labelled as having acute fatty liver of pregnancy, HELLP syndrome (Haemolysis, Elevated Liver enzymes, Low Platelet count), haemophagocytic lymphohistiocytosis (HLH) or macrophage activation syndrome, all rare occurrences in pregnancy with clinical and laboratory features that overlap with acute HSV hepatitis.

More recent reports have expanded the spectrum of risk to include elderly patients [28], individuals with cirrhosis, postoperative patients, and those with critical illness, all of whom may exhibit transient or localized immune dysfunction [15, 28, 29, 30, 31]. These observations suggest that susceptibility to HSV hepatitis is not binary but exists along a continuum of immune competence. Importantly, HSV hepatitis should be considered in any patient with acute hepatitis or acute liver failure, regardless of whether overt immunosuppression is present.

In HSV hepatitis, history taking may provide subtle but decisive clues when clinical signs are nonspecific. For instance, in Case 1, the absence of mucocutaneous lesions could have easily diverted attention away from HSV infection. However, the history of recent sexual exposure to a partner with active herpes labialis, combined with a prior bone marrow transplant, raised early suspicion and prompted virological testing. In Case 2, disclosure of recent initiation of sexual activity in a patient receiving immunomodulatory therapy was critical to reframing the diagnosis from drug‐induced liver injury to disseminated HSV infection. Importantly, history taking should extend beyond medical diagnoses to include sexual history, recent exposures, travel, procedures, and daily activities. Failure to elicit these details may delay diagnosis and initiation of antiviral therapy, directly impacting survival. A focused but comprehensive history—including sexual exposure and recent HSV symptoms in partners—should be systematically obtained in patients with unexplained acute hepatitis.

HSV hepatitis typically presents as disseminated HSV infection and, often, as a systemic inflammatory syndrome rather than isolated liver disease. However, clinical manifestations are variable and frequently nonspecific, contributing to diagnostic delay. Fever is the most common presenting symptom, followed by right upper quadrant or diffuse abdominal pain, nausea, vomiting, and anorexia [15, 16]. Classical herpetic skin or mucosal lesions are absent in more than half of cases, and their absence should not dissuade clinicians from considering the diagnosis [3]. Patients are often anicteric despite severe hepatocellular injury, and hepatic encephalopathy may be present early in fulminant cases. Laboratory findings typically reveal striking elevations in aminotransferases—often exceeding 1000 times the upper limit of normal—with relatively normal bilirubin levels. Leukopaenia, thrombocytopaenia, coagulopathy, disseminated intravascular coagulation, acute kidney injury, and features of septic shock are frequently observed [10, 16].

The cases presented illustrate this variability. Case 1 manifested with acute urinary retention, likely reflecting HSV‐related autonomic or sacral plexus involvement. Case 2 demonstrated disseminated cutaneous involvement at atypical sites, including palms and soles. Case 3 presented with severe mucositis and ocular involvement, which provided an early diagnostic clue. Thus, HSV hepatitis should be suspected in patients with acute “anicteric” hepatitis, systemic inflammatory features, and cytopenias, even in the absence of cutaneous lesions.

The diagnosis of HSV hepatitis is frequently delayed due to its rarity and lack of pathognomonic clinical or imaging features. In the review by Norvell et al., HSV hepatitis was clinically suspected in only 22.6% of cases, with more than half diagnosed post‐mortem [2]. Liver biopsy remains the histopathological gold standard, demonstrating hemorrhagic or coagulative necrosis with viral cytopathic effects, including enlarged ground‐glass nuclei and marginal chromatin [15, 28, 31]. However, biopsy is often contraindicated in patients with coagulopathy or haemodynamic instability, which are common in acute liver failure. Imaging modalities such as ultrasound and computed tomography are nonspecific but may reveal hepatomegaly or multiple small hypodense lesions corresponding to necrotic foci [10]. These findings are not diagnostic and overlap with other infectious or infiltrative processes. The advent of PCR‐based HSV DNA detection in blood and tissue has transformed diagnostic practice. Detection of HSV DNA in plasma provides a rapid, sensitive, and minimally invasive diagnostic tool that can support early therapeutic decisions [15, 16]. Serology has limited utility in acute settings and may be misleading, particularly in reactivation or in immunocompromised patients.

A further point that merits explicit consideration is the occurrence and clinical significance of HSV DNAemia. HSV is fundamentally a neurotropic virus that establishes latency in sensory ganglia and typically replicates locally at mucocutaneous sites [3, 4]. Accordingly, detection of HSV DNA in plasma should be interpreted as a marker of pathological systemic spread. Therefore, HSV DNAemia reflects a failure of early viral containment, occurring in the setting of overwhelming viral replication and/or impaired innate and adaptive immune responses [7, 8, 9].

In immunocompromised patients, defective cell‐mediated immunity permits uncontrolled reactivation and spread of HSV. Indeed, impaired CD8^+^ T cells fail to recognize and eliminate infected cells due to reduced cytotoxic granule release (perforin and granzymes) and diminished secretion of antiviral cytokines such as Interferon (IFN)‐γ. Concomitantly, weakened type I IFN‐α/β signalling [21] compromises early intracellular antiviral states by failing to upregulate genes that inhibit viral transcription and replication. In line with this, a recent retrospective study on the prevalence of type I IFN‐neutralizing autoantibodies showed that over a third of patients HSV‐induced fulminant hepatic failure (FHF) had such antibodies compared with none with FHF caused by HAV and HBV, emphasizing their pathogenetic role as major determinant in this specific setting [32]. In parallel, reduced natural killer (NK) cell activity limits early killing of infected cells lacking adequate MHC class I expression, a key HSV immune evasion target [8]. Taken together, these defects allow enhanced viral replication at mucocutaneous sites, facilitate cell‐to‐cell spread, and increase the likelihood of viremia and dissemination to visceral organs [33]. In this context, high levels of circulating HSV DNA, as observed in our cases, may represent a clinically meaningful indicator of host–virus disequilibrium and severe disseminated disease. Beyond its diagnostic utility, HSV DNAemia may therefore have prognostic relevance, identifying patients at increased risk of rapid clinical deterioration and underscoring the need for urgent initiation of antiviral therapy.

In all three cases presented, diagnosis was established without liver biopsy, relying on clinical suspicion supported by molecular virology. This approach reflects current best practice. HSV PCR testing should be promptly performed in patients with unexplained acute hepatitis or acute liver failure, but treatment should not be delayed while awaiting results.

The prognosis of HSV hepatitis is strongly dependent on the timing of antiviral therapy initiation. Without treatment, mortality approaches 90%, whereas early initiation of intravenous acyclovir significantly improves survival and reduces the need for liver transplantation [11, 12]. Empiric intravenous acyclovir (10 mg/kg every 8 h, adjusted for renal function) is recommended in patients with suspected HSV hepatitis or acute liver failure of unknown aetiology, particularly in high‐risk settings such as pregnancy, transplantation, or immunosuppression [13, 28, 34]. Acyclovir resistance, primarily due to thymidine kinase mutations, remains uncommon but is increasingly reported in immunocompromised populations [35, 36]. Foscarnet or cidofovir represent effective alternatives in cases of acyclovir resistance or inadequate virological response, and combination therapy may be considered in patients with extremely high viral loads or progressive disease, as illustrated in Cases 1 and 3 [1, 35, 36].

When HSV hepatitis progresses to fulminant liver failure, urgent liver transplantation may be lifesaving but is associated with high rates of recurrent HSV infection and limited long‐term survival, with reported one‐year survival around 40% [12, 14]. Long‐term or lifelong antiviral suppression is generally recommended following recovery. The European Association for the Study of the Liver Clinical Practice Guidelines on acute (fulminant) liver failure explicitly recommend high‐dose intravenous acyclovir in neonates and consideration of empiric therapy in adults until HSV infection is excluded [12].

Prevention of HSV hepatitis is difficult, but there are feasible strategies in the context of immunosuppressed transplant recipients. HSV infection in such patients can rarely be derived from the donor organ, usually resulting in acute liver failure within 4 days of transplantation. It has been suggested that universal recipient HSV serology should be performed pre‐transplant, and if negative, in those not receiving valganciclovir prophylaxis for CMV infection, then appropriate prophylaxis with acyclovir should be initiated [37, 38].

## 5 Conclusion

HSV hepatitis remains a rare but devastating cause of acute hepatitis and acute liver failure. Its clinical importance lies not in its frequency but in its fulminant course, high mortality when unrecognized, and the narrow therapeutic window during which antiviral therapy can be lifesaving [35, 39]. As illustrated by the present case series, HSV hepatitis can occur across a broad spectrum of patients, ranging from profoundly immunocompromised individuals to those without overt immune dysfunction.

The absence of mucocutaneous lesions, nonspecific systemic presentation, and frequent misattribution to drug‐induced or ischemic hepatitis continue to delay diagnosis. A characteristic laboratory pattern—marked cytolytic enzyme elevation with minimal cholestasis, often accompanied by cytopenia and coagulopathy—should prompt consideration of HSV infection. Empiric antiviral therapy should not be delayed when HSV hepatitis is a reasonable diagnostic possibility, as early intravenous acyclovir is strongly associated with improved survival. Thus, in patients with acute “anicteric” hepatitis and systemic inflammatory features, HSV infection should be actively excluded—and empiric intravenous acyclovir initiated—regardless of whether overt immunosuppression is immediately apparent.

## Author Contributions

Conceptualization: M.U.M., S.L., A.D.M., W.I.; writing – original draft: M.U.M., W.I.; writing – review and editing: M.U.M., W.I., G.Ö.Ş, A.D.M. S.L. Supervision: M.U.M, S.L., W.I., G.Ö.Ş.

## Funding

The authors have nothing to report.

## Conflicts of Interest

The authors declare no conflicts of interest.

## Data Availability

The data that support the findings of this study are available on request from the corresponding author. The data are not publicly available due to privacy or ethical restrictions.

## Appendix A

ESGVH Members: Mario U. Mondelli, Gülşen Özkaya Şahin, William Irving.
